# Continuous Ketone Monitoring: Data From a Randomized Controlled Trial

**DOI:** 10.2196/85548

**Published:** 2026-01-13

**Authors:** Simon K Kjær, Lukas Ochsner Reynaud Ridder, Mads Svart, Nikolaj Rittig, Lise Nørkjær Bjerg, Birgitte Sandfeld-Paulsen, Henrik Holm Thomsen

**Affiliations:** 1 Steno Diabetes Center Aarhus, Aarhus University Hospital, Aarhus, Denmark; 2 Department of Clinical Medicine, Aarhus University, Aarhus, Denmark; 3 Department of Endocrinology and Internal Medicine, Aarhus University Hospital, Aarhus, Denmark; 4 Department of Internal Medicine, Regional Hospital Horsens, Horsens, Denmark; 5 Department of Clinical Biochemistry, Regional Hospital Central Jutland, Viborg, Denmark; 6 Department of Clinical Biochemistry, Aalborg University Hospital, Aalborg, Denmark; 7 Medical Diagnostic Center, University Clinic for Innovative Patient Pathways, Regional Hospital Central Jutland, Heibergs Allé 4K, Viborg, 8800, Denmark, 45 78447000

**Keywords:** continuous ketone monitoring, exogenous ketosis, beta-hydroxybutyrate, ketone sensor technology, ketone ester

## Abstract

In our study, a commercially available continuous ketone monitoring device captured β-Hydroxybutyrate (BHB) dynamics during exogenous ketosis but revealed a gradual decline day-to-day BHB concentrations over 14 days in both ketone ester and placebo groups, likely reflecting sensor drift.

## Introduction

Continuous measurement of ketone bodies is of scientific and clinical interest, providing insights into type 1 and type 2 diabetes, ketogenic diets, intermittent fasting, and exogenous ketone precursor supplementation. Current finger-prick point-of-care testing (POCT) devices are invasive, intermittent, and fail to capture dynamic fluctuations [1]. Continuous ketone monitoring (CKM), a small device measuring interstitial ketone (β-hydroxybutyrate, BHB) levels, offers a potential solution [2]. CKM research, however, remains in its early stages, with only a single commercially available device at present (SiBio KS1, Hong Kong), to the best of our knowledge. Exogenous ketone supplementations are currently studied for potential therapeutic applications, including weight loss, enhanced exercise performance, and the management of neurodegenerative, cardiovascular, and inflammatory conditions [3-5]. We hypothesized that CKM would accurately track BHB and evaluated its performance under sustained intermittent supraphysiological ketosis.

## Methods

### Study Design

This work is part of a larger study on exogenous ketosis and erythropoiesis (Thomsen et al, unpublished). CKM became available midway through the study and was therefore applied sequentially in the final 7 of the 16 healthy volunteers. Participants were randomized to receive either a ketone ester (KE) drink (500 mg/kg/d) or a placebo (PBO), matched for volume, taste, and viscosity. Over two weeks, drinks were consumed two to three times daily, with half the dose before sleep. Participants were blinded to CKM readings, while investigators were not blinded. We tested the effects of time, treatment, and their interaction on log-transformed BHB area under the curve (AUC) using a linear mixed-effects model and applied polynomial contrasts to assess linear trends.

### Ethical Considerations

The study was conducted in accordance with the Declaration of Helsinki II, approved by the regional ethics committee (#1-10-72-221-22), and registered with ClinicalTrials (NCT06053138). Oral and written informed consent was obtained from all participating patients. Participant data were pseudonymized to ensure confidentiality. Participants received financial compensation for their time and participation.

## Results

**Figure 1.**
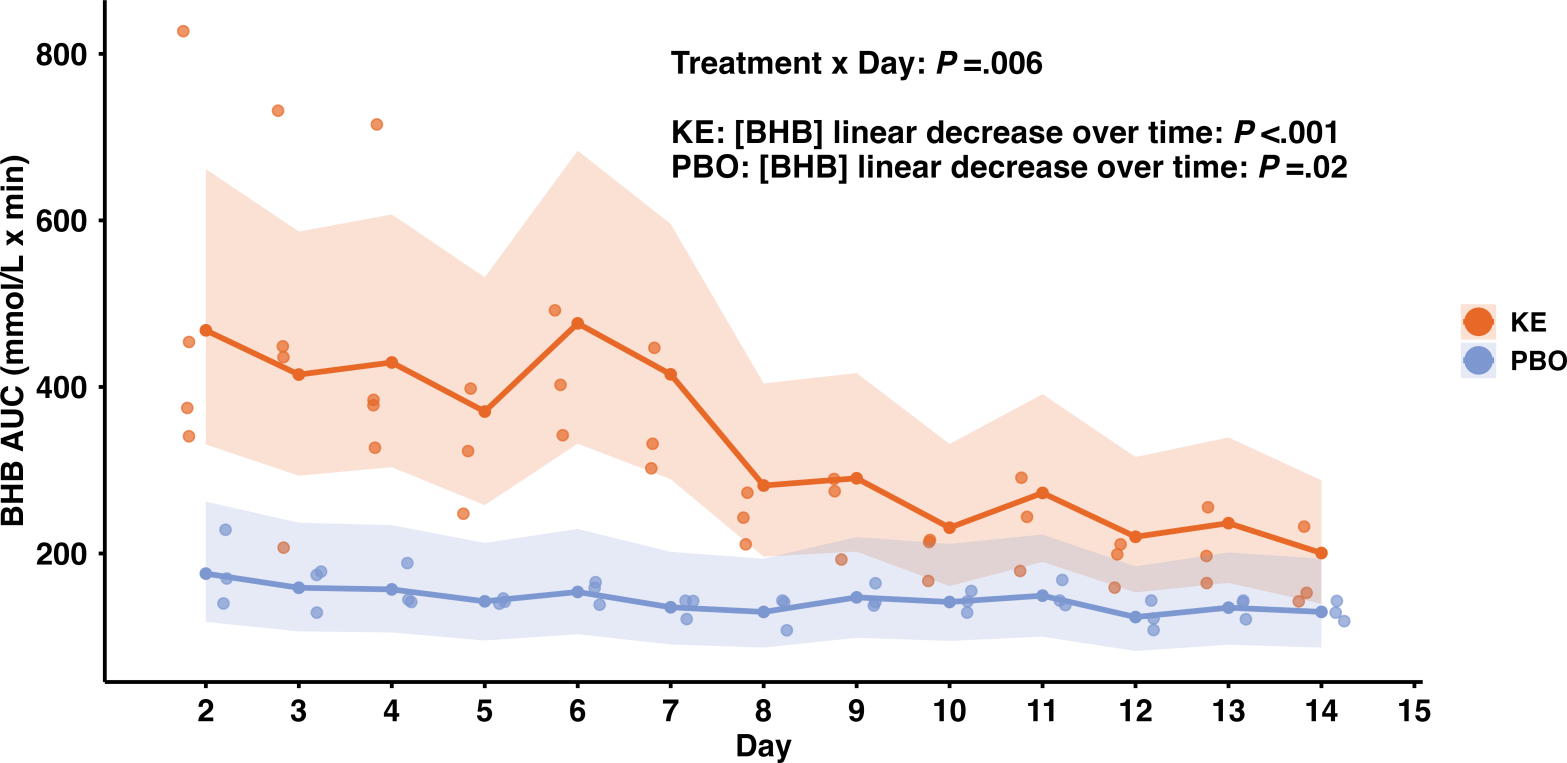
Day-by-day changes in total BHB area under the curve (AUC) for both the Ketone Ester (KE) group (n=4, orange) and placebo (PBO) group (n=3, blue). Scatter points represent individual AUC measurements for each participant across the 14 study days. Solid lines depict the back-transformed least-square means of BHB concentrations from a mixed-effects model, estimated separately for each day and treatment group, and the shaded regions represent the confidence intervals. BHB: beta-hydroxybutyrate.

A total of 7 participants wore CKM devices: 4 in the KE group (3 female, 1 male) and 3 in the PBO group (2 female, 1 male). Median age was 41 years (IQR 28–55). One KE participant’s sensor detached on day 4 and was not replaced, but CKM readings until detachment were included in the analyses. BHB AUCs were significantly influenced by both day and treatment, with an interaction effect (*P*=.006). In the KE group, BHB showed a significant linear decrease over 14 days (*P*<.001), and a smaller but significant decline was also observed in the PBO group (*P*=.02). Consequently, group differences diminished, with KE and PBO becoming indistinguishable by the final day (Figure 1).

## Discussion

This study evaluated the performance of a commercially available CKM device during 14 days with intermittent exogenous ketone supplementation. Our findings demonstrate that the CKM detected increases in interstitial BHB concentrations following KE ingestion but revealed a progressive decline in BHB concentrations over the 14-day study period in the KE group, indistinguishable from the PBO group on the last study day. This contrasts with two prior studies in which participants received KE for 14 days before ingesting 25 g KE in a laboratory setting on day 15 [6,7]. In those studies, peaks reached ~2.3 mM at 1 hour and declined to ~0.5 mM at 4 hours, with no evidence of a declining peak BHB concentration following a comparable period of intermittent exogenous ketosis. Importantly, we observed a temporal decline in BHB concentrations also in the placebo group, highly suggesting a ketone-independent physiological or measurement-related drift. Therefore, this raises the possibility of sensor-related limitations. Potential explanations include sensor enzyme degradation, biofouling, temperature effects, compression, or interstitial variability [8]. The underlying sensor principle is not fully disclosed but thought to use a modified electrochemical method reacting selectively with BHB in interstitial fluid. In comparison, an in-development multianalyte sensor using a three-electrode system with NAD^+^-dependent β-hydroxybutyrate dehydrogenase and osmium-based redox chemistry has shown stable 14-day performance in 12 healthy, low-carbohydrate-consuming participants [9,10]. A future study is anticipated with interest since it will assess the accuracy of the same device used in our study, SiBio KS1, in subjects following a 14-day ketogenic diet (NCT06420518). Limitations for our study include not comparing the CKM-derived ketone levels with gold standard blood BHB measurements (eg, finger-prick tests), making it difficult to definitively decide if our observations are due to sensor-specific limitations or not. Additionally, the small sample size and statistical power may impact the generalizability of our findings, and it is important to note that the study was not originally designed to evaluate CKM performance.

In conclusion, CKM captured BHB dynamics during exogenous ketosis but revealed a gradual decline in day-to-day BHB AUC over 14 days in both KE and PBO groups, likely reflecting sensor drift rather than physiological adaptation. Larger controlled studies with direct comparison of CKM and blood BHB measurements are needed to confirm accuracy and clinical utility, and must include more than a single batch of CKM devices.

## Abbreviations

AUC: area under the curve
KE: ketone ester
BHB: β-hydroxybutyrate
CKM: Continuous ketone monitoring
PBO: placebo
POCT: point-of-care testing
